# Editorial: Halophytes: salt stress tolerance mechanisms and potential use

**DOI:** 10.3389/fpls.2023.1218184

**Published:** 2023-06-22

**Authors:** Raoudha Abdellaoui, Amr Elkelish, Ali El-Keblawy, Hedi Mighri, Fayçal Boughalleb, Esmaeil Bakhshandeh

**Affiliations:** ^1^ Laboratory of Rangeland Ecosystems and Valorization of Spontaneous Plants LR16IRA03, Arid Regions Institute, University of Gabès, Médenine, Tunisia; ^2^ Biology Department, College of Science, Imam Mohammad Ibn Saud Islamic University (IMSIU), Riyadh, Saudi Arabia; ^3^ Botany and Microbiology Department, Faculty of Science, Suez Canal University, Ismailia, Egypt; ^4^ Department of Applied Biology, College of Sciences, University of Sharjah, Sharjah, United Arab Emirates; ^5^ Genetics and Agricultural Biotechnology Institute of Tabarestan & Sari Agricultural Sciences and Natural Resources University, Sari, Iran

**Keywords:** soil salinization, osmotic stress-associated traits, biochemical adaptation, phenotypic plasticity and resilience, salinity stress alleviation, biosaline agriculture

## 1 Introduction

Drought and salinity are among the principal environmental factors restricting plant growth, reducing productivity in numerous plant species. Both rising sea levels and increasing temperature contribute to this salinization; a common problem predicted to become more widespread worldwide. About 3.6 billion of the 5.2 billion hectares of arid lands used for agriculture suffer from erosion, salinization, and soil degradation (Riadh et al., 2010).

In addition, climate change over the coming decades is expected to decrease annual precipitation in semi-arid, arid, and desert regions. As a result, good quality water would be increasingly reserved for urban and drinking purposes, so brackish and saline water could be an alternative for irrigation (Shabala, 2013). Moreover, it is estimated that by 2050, the world population will increase to 9.7 billion, which would necessitate more than 70% of food production, causing additional pressure on the arable lands (Calone et al., 2021).

To achieve such goal the salt-affected, degraded, and marginalized regions should be valorized and used as promising areas for valuable resources, especially in arid lands. The restoration and rehabilitation of salt-affected lands for crop production can be achieved through several methods, including halophytes and extremophiles that respond to salt stress factors in the short term by complex accommodation processes and in the long term by developing adaptation strategies.

Halophytes have strong potential for developing saline agriculture strategies and could later improve the natural ecosystem. These plants are adapted to growing in high-salt environments; they have unique mechanisms that allow them to survive and thrive in extreme saline conditions. Planting halophytes in salt-affected areas can improve soil quality, restore biodiversity, produce valuable products, such as animal feeds and renewable energy sources, and save freshwater, scarce depleted natural resources. They have been used successfully to restore wetlands, salt marshes, and other coastal habitats. Halophytes capable of growing and reproducing in extremely saline conditions could depict a treasure trove of genes used to improve the production of crops in saline lands and to develop biosaline agriculture (Cheeseman, 2015; Ventura et al., 2015).

This Research Topic is to promote biosaline agriculture and maximize the value of their products by identifying relevant traits that can be used to understand and investigate halophytes’ salt tolerance mechanisms. Furthermore, despite knowledge gaps, exploring the genetic potential of halophytes seems an important tactic to enlighten on the adaptive mechanisms and unveil the threshold environment of halophytes for survival and strengthen plant productivity under saline conditions.

In this special Research Topic of Frontiers in Plant Science, the articles concentrated on three main themes: (1) mechanisms and strategies for adaptations to salinity, (2) alleviation of salinity stress, and (3) sustainable utilization of halophytes for restoration of salt-affected lands.

## 2 Mechanisms and strategies of plant adaptations to salinity

### 2.1 Metabolomic approaches

Plants adopt several strategies for survival the salt-affected lands. For example, the multipurpose tree *Gliricidia sepium* adopts a salt-excluding strategy through leaf defoliation and limiting salt absorption in the roots. Besides, the metabolomics analysis helped postulate that lignin accumulation in the cell wall, accumulation of some phytosterols that adjust membrane structure and characteristics, and lysine biosynthesis play a role in promoting the salinity adaptation response (Braga et al.). In another article, morpho-physiological approaches revealed the main survival strategy of the Semi-mangrove *Clerodendrum inerme* to salinity through reshaping its metabolic and ion profiles. Besides, this species adapted to salinity stress at the seedling stages by altering its metabolism of nucleotides, enzymes, transcription factors, amino acids, plant hormones, and carbohydrates. By rebalancing the energy allocation between growth and stress tolerance, *C. inerme* moderates its development rate to withstand both short- and long-term salt adversity (Liang et al.).

### 2.2 Molecular and genomic approaches

Salinity stress is a major environmental factor that negatively affects plant growth and productivity. In response to salt stress, plants undergo a range of physiological, molecular, and genomic changes. Molecular and genomic approaches have been employed to study the mechanisms underlying the response of plants to salinity stress.

Wang et al. investigated *Sesuvium portulacastrum*’s salt tolerance mechanism. RNA-Seq identified DEGs in salt-stressed plant roots and leaves. Na is taken in by cyclic nucleotide-gated channels (CNGCs), whereas Na^+^/H^+^ exchangers (NHXs) push it out and store it. Glutathione metabolism scavenged reactive oxygen species and osmoprotected soluble sugar and proline.

On the other hand, Jiang et al. studied *Chenopodium quinoa*, which is categorized as a halophyte with exceptional nutritional qualities. Quinoa had 26 CqCrRLK1L and 18 CqRALF genes. After salt treatment, three CqCrRLK1L genes were substantially up-regulated by transcriptomic profiling. Biochemical analysis showed that CqRALF15 physically interacts with CrRLK1L, CqFER, and AtFER proteins. Arabidopsis overexpressing CqRALF15 bleaches leaves more under salt stress.

The molecular and genomic approaches on economic crops are fulfilled in two successive years by evaluating 30 cotton genotypes under drought and/or heat stresses. Results showed that all the morphological and quality characteristics, for instance, boll weight, fiber fineness, fiber strength, and fiber length are significantly decreased under drought and heat. However, superoxide dismutase (SOD), ginning out turn percentage (GOT %), and flavonoids increased particularly (Farooq et al.).

Finally, Rice, a salt-sensitive plant, was extensively studied by Wang et al. and using a Genome-wide association study (GWAS). They identified 65 Quantitative Trait Locus (QTLs) associated with salt tolerance. They found that LOC_Os06g47970, LOC_Os06g47820, LOC_Os06g47720, and LOC_Os06g47850 were found to be related to salinity stress. They suggested that these candidate genes are helpful in rice breeding programs.

Overall, molecular and genomic approaches have provided valuable insights into the mechanisms underlying the response of plants to salt stress. These approaches have identified key genes and pathways that are involved in response to salt stress, which could be targeted to improve plant tolerance to salinity stress.

### 2.3 Alleviation of salinity stress

Several types of phytohormones and endophytic microbes, and arbuscular mycorrhizal fungi can help plants alleviate salinity stress. Jasmonic acid (JA), a phytohormone, alleviated salinity stress in wheat by regulating morphological, biochemical, and genetic attributes (Sheteiwy et al.). The authors demonstrated that JA modulates the salinity effect in wheat plants through different pathways, including improving ion homeostasis that reduces ion toxicity, recovering chloroplast ultrastructure that increases the photosynthesis efficiency, and stimulating antioxidant defense machinery that scavengers ROS accumulation. In another study, Al-Elwany et al. reported that the foliar spraying folic acid (FA) mitigated the salinity effect in the medicinal plant *Plectranthus amboinicus*. FA played a key role in enhancing photosynthetic efficiency, pigment contents, leaf osmoprotectant compounds, and the concentrations of soluble sugars, free amino acids, proline, and total phenolics in *Plectranthus amboinicus* plants subjected to salinity stress. Such improvement in the physiological and biochemical attributes improved the growth and production of essential oil content of salt-stressed plants.

In an interesting approach, Selim et al. used magnetically treated seawater to reduce the salt effect on wheat plants. Such treatment improved wheat’s seed germination, growth, and yield attributes under saline conditions by modulating physiological and anatomical attributes. Wheat irrigated with magnetic-treated seawater improved leaf water deficit, abscisic acid content, and transpiration rate. Irrigation with seawater treated with a magnetic field is a promising way to mitigate the salinity effects. However, further studies are needed to explore possible mechanisms behind this technology.

## 3 Sustainable utilization of halophytes

The salinization of soil and water severely restricts sustainable agricultural development in semiarid and arid regions. Halophytes irrigated with seawater and cultivated in salt-affected soils could be a promising solution for freshwater scarcity, soil salinization, and fodder shortages. For example, Wang et al. assessed the forage nutritional value and salt removal capacity of the halophyte *Salsola salsa* under varied salinity levels in the arid lands of China. The researchers found that salinity increased the crude protein content and mineral nutrition and decreased the fiber concentration, increasing the forage nutritional value of *S. salsa*. Interestingly, this halophyte can bioconcentrates salts in its tissues, rehabilitating salt-affected soil. The study concluded that *S. salsa* could be a potential crop in saline soils irrigated with brackish water. In addition, it provides a possible additional source of fodder in arid regions where non-saline arable lands and freshwater are limited.

Moreover, Abideen et al. reviewed potential halophytes and algal species as alternatives for food biomass used in producing renewable energy sources. The review summarizes the pitfalls and precautions for producing biomass in an environmentally friendly way that does not harm coastal ecosystems. Several species with great potential as sources of bioenergy were highlighted. Despite the tremendous advantages of their use as non-food biomass for producing renewable energy, some negative impacts are associated with their introduction in new environments, such as ecosystem alteration, the introduction of invasive species, and transmissible plant diseases.

Furthermore, in an interesting systematic review article, Ashilenje et al. studied crop species mechanisms and ecosystems services for sustainable cropping systems in salt-affected arid regions using data obtained from the results of 20 studies published in peer-reviewed journals between the years 2000 and 2021. This paper examined the mechanisms and synergies of species that can be incorporated into cropping systems to alleviate soil salinity problems and maintain forage productivity in dry lands. Clearly, halophyte and non-halophyte forages have converging mechanisms of salinity tolerance manifested through increased photosynthesis and productivity. Indicators of the ubiquity of ecosystem services of both halophyte and non-halophyte species exist along a continuum soil salinity increase. Possible synergies among perennials, halophytes, herbaceous plants, fungi, and bacteria to combat the effects of soil salinity have been identified. These synergies can be used to develop sustainable forage cropping systems that improve saline soils and nutrient cycling to maintain optimal forage productivity.

Besides, more research is needed to overcome seed availability, germination, dormancy problems, and propagation techniques. In this context, Chen et al. assessed the germination requirements (e.g., light and temperature), salinity, and drought tolerance of naked and winged (i.e., with the attached perianth structures) seeds of several *Salsola* species. Detailed germination information was provided for cultivating four *Salsola* species in degraded saline soils.

In perspective, we believe that this Research Topic highlights interesting avenues for using halophytes as food, feed, and source of secondary metabolites and to understand better how we can improve crops’ tolerance to saline stress.

## Author contributions

All authors listed have made a substantial, direct, and intellectual contribution to the work and approved it for publication.
